# Material Efficiency and Circularity Goals to Achieve a Carbon-Neutral Society by 2050

**DOI:** 10.1021/acs.est.4c08719

**Published:** 2025-03-20

**Authors:** Sho Hata, Keisuke Nansai, Yosuke Shigetomi, Minami Kito, Kenichi Nakajima

**Affiliations:** † Social Systems Division, National Institute for Environmental Studies, 16-2 Onogawa, Tsukuba 305-8506, Japan; ‡ Graduate School of Frontier Sciences, The University of Tokyo, 5-1-5 Kashiwanoha, Kashiwa, Chiba 277-8563, Japan; § Material Cycles Division, National Institute for Environmental Studies, 16-2 Onogawa, Tsukuba 305-8506, Japan; ⊥ College of Science and Engineering, Ritsumeikan University, 1-1-1 Nojihigashi, Kusatsu 525-8577, Japan; # Faculty of Socio-Environmental Studies, Fukuoka Institute of Technology, 3-30-1 Wajirohigashi, Higashi-ku, Fukuoka 811-0295, Japan

**Keywords:** input−output analysis, material footprint, material efficiency, circular economy, scenario analysis

## Abstract

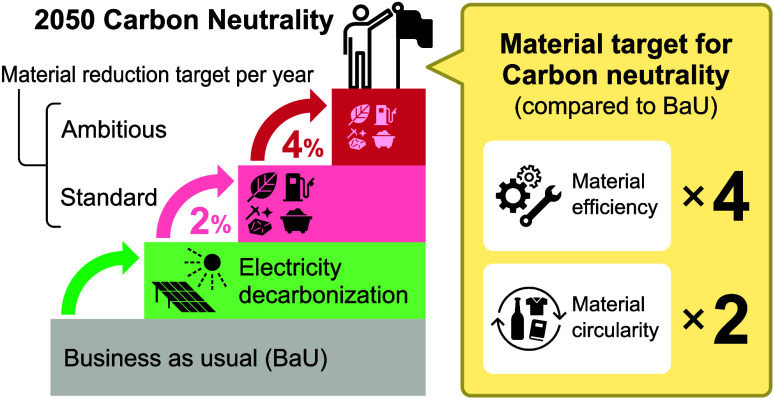

Decarbonizing materials production presents a major challenge to achieving a carbon-neutral society. The current carbon-neutral roadmap for materials generally assumes an uncertain dissemination of innovative production technologies, making the changes in material flows required for a carbon-neutral society unclear. This study introduces three material reduction scenarios and an input–output optimization model to explore material flows in Japan for achieving carbon neutrality by 2050 without disseminating innovative technologies. The results indicate that Japanese carbon emissions could reach neutrality by 2050 if the country can manage a yearly material reduction of 4% together with the decarbonization of electricity generation. To achieve this, the material efficiency of consumption must increase nearly 4-fold, and the material circulation rate must be doubled. Achieving such a rapid improvement in material efficiency and circularity is as challenging as the early large-scale deployment of material decarbonization technologies. Therefore, the critical choice in the remaining years is whether to bet heavily on innovative material decarbonization or prioritize technologies and policies accelerating material efficiency.

## Abstract 2

Achieving a carbon-neutral society will require technologies and policies that not only focus on the development of innovative material decarbonization technologies but also increase resource efficiency and circularity many times over.

## 1 Introduction

The Sixth Assessment Report (AR6) issued by the IPCC emphasized that to meet the climate goals set out in the Paris Agreement, it will be necessary to reach net-zero greenhouse gas (GHG) emissions, that is, carbon neutrality, by 2050 in order to keep temperature rise within the 1.5° target.^1^ However, the latest emission gap report^2^ revealed that global GHG emissions increased by 1.2% from 2021 to 2022 to reach a record of 57.4 gigatons of CO_2_ equivalent (Gt CO_2_e), exceeding 2019 (pre-COVID-19 pandemic) levels. To meet the climate targets, countries will need to continually reassess their GHG emission reduction goals and effectively manage their emission levels.^3−5^

Material use is a significant trigger of GHG emissions,^6−8^ with emissions from material production estimated to have accounted for a quarter of global emissions in 2015.^9^ The troublesome point is that emissions for material production are difficult to electrify due to fuel consumption for oxidation–reduction and chemical reactions. Hence, the decarbonization of material use cannot be achieved solely by shifting power sources to renewable energy.

The International Energy Agency (IEA) was quick to release a roadmap for achieving global net-zero-emissions.^10,11^ This report can be regarded as a case study illustrating the requirements for achieving a net-zero society based on the harnessing of innovative technologies. With specific regard to emissions from materials production, emission reductions in steel and cement will be accomplished through hydrogen and carbon capture, utilization, and storage (CCUS) technologies, which, respectively, account for roughly 10 and 40% of total emission reductions from materials production. However, these are early-stage technologies still in the prototype development phase.

While it is believed by many that the decarbonization of materials production can be realized through the widespread introduction of innovative technologies,^10,11^ such technologies not only face technical challenges but also suffer from a lack of thorough consideration of the economic policy instruments required to adopt such high-cost technologies.^12^ In other words, it is quite possible that these technologies will not be widely available by 2050.

Other than relying on the development of innovative material technologies, the complementary way to mitigate the GHG emissions associated with material production is to manage material use, including the demand-side measures that can avoid unreasonably large requirements for negative emission technologies and have multiple cobenefits.^13,14^ Economy-Wide Material Flow Accounting (EW-MFA)^15−18^ is a powerful tool for understanding and managing material flows at the national level, providing indicators of material management such as resource productivity and cyclical use rates, which have been used in Europe^19^ and elsewhere for the design of a Sound Material-Cycle Society^20^ and a circular economy (CE).^21,22^ Japan has defined future target values for four such indicators in accordance with the fundamental plan defined by the law and is using them to help manage the nation’s material flows.^23^ Furthermore, material flow improvements contribute to reducing carbon emissions; in concrete terms, material efficiency increases, such as more intensive use, lifetime extension, and less material design in housing and cars, have the potential to reduce lifecycle GHG emissions by 35% for houses and 40% for cars.^7,24,25^ However, it remains unclear to what extent economy-wide material flows will need to be transformed by increasing material efficiency to achieve a carbon-neutral society by 2050. This prevents us from envisioning material flows in a carbon-neutral society or establishing consistent future goals for appropriate material flow indicators.

To fill this gap, this study aims to explore the structural changes in material-waste-cyclical flows needed to reach net-zero carbon emissions by 2050. Here, we focus in particular on the effect of material reduction and circulation on the direct and indirect carbon emissions of consumption and consider how to maintain the current commodity consumption patterns as much as possible. Specifically, we set four future scenarios with different material reduction rates and strategies for material efficiency improvement and developed an optimization model using Environmentally Extended Input–Output (EEIO) analysis. The target country is Japan, which has legal material flow indicators^23^ and the fifth highest GHG emissions in the world.^26^ The model optimizes material flows in Japan from 2015 to 2050 by minimizing the change in commodity consumption patterns under constraints on the rate of reduction in total material input.

## 2 Methods and Data

### 2.1 Overview of Our Model

We first developed a model and a data set to calculate the material footprint (MF) and carbon footprint (CF), which represent the domestic material use and GHG emissions induced by final demand, respectively (Section 2.2). In our model, MF and CF are calculated based on domestic household consumption, exports, and the fixed capital required to produce these goods and services.

Next, we constructed an optimization model designed to minimize changes in commodity consumption (million Japanese yen) while achieving reduction targets for material use, calculated as MF, with the ultimate goal of achieving carbon neutrality (Section 2.3). Four scenarios were defined, each with different material reduction targets and material efficiency improvement strategies (Section 2.4). Optimization was performed every 5 years from 2015 to 2050. We assume that keeping household consumption as close as possible to its current state is the most understandable for interpreting the results and is relatively acceptable to most consumers. Therefore, the optimization explored material flows that achieve carbon neutrality with minimal change in household consumption.

### 2.2 Definition of Material and Carbon Footprint Using the Capital Endogenized IO Model

The methodology for the footprint calculation employed in this study is based on Hata et al.,^27^ which estimated the capital-embodied material and carbon footprints associated with Japanese household consumption and export in 2015. To calculate capital-embodied footprints, capital endogenization methods—both the augmentation method and the flow matrix method—have been used for decades.^28^ In recent years, with the expansion of databases for capital flow matrices and methodologies, the flow matrix method has been widely used to endogenize either gross fixed capital formation (GFCF) or consumption of fixed capital (CFC).^29−33^ In this study, to incorporate changes in both GFC and CFC in the scenario analysis, we adopt the matrix-augmentation method proposed by Hata et al.,^27^ which uses a capital matrix to endogenize both GFCF and CFC.

The system boundary of the study is limited to a single nation; the spillover effects of imported materials (imported resources and products) are traced back only to the point of import. Therefore, when the MF induced by domestic final demand and exports is calculated using this model, it corresponds to the domestic material input as defined by the EW-MFA. In addition, the model includes data that allow the calculation of the domestic cyclical use of materials as a footprint.^27,34^ As a result, this study defines MF as the domestic material input and cyclical use of material induced by final demand, which differs from the definition commonly used in the literature: “the global allocation of used raw material extraction to the final demand of an economy”^35^. The reason for setting such a system boundary is that Japan’s material flow targets^23^ are defined on the basis of total material input (the sum of domestic material input and cyclical material use). Details of the material flow targets are listed in Section 2.4. In this study, the model is constructed to match the boundaries of these material flow targets.

Here, **F**_R,(*t*)_, the capital-embodied MF of natural resources (*k*) at a year *t* (*t* = 2015, 2020, 2025, 2030, 2035, 2040, 2045, 2050), is defined in eq 1 as follows
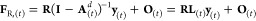
1where **L**_(*t*)_ = (**I** – **A**_(*t*)_^*d*^)^−1^ is the Leontief inverse matrix. Matrix  is composed of input coefficients including the endogenized fixed capital effects, excluding the spillover effects of imports in year *t*. Matrix **B**_(*t*)_ and **C**_(*t*)_ represent the capital formation matrix and the capital utilization matrix in year *t*, respectively (see our previous work^27^ for more information regarding endogenous fixed capital formation and utilization effects). Each element *m*_*i*_ of vector  represents the import ratio of commodity *i*.  is a matrix, with elements *r*_*kj*_ representing the direct input of natural resource *k* per unit production in sector *j*; vector  represents the final demand for commodity *i*;  is a matrix of the direct input of material *k* to sector *j* of final demand.

The capital-embodied MF of cyclical use (*s*) in year *t*, **F**_U,(*t*)_, is estimated from the input of natural resources using eq 2
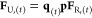
2where  represents the generation rate of cyclical use *s* in response to the input of natural resource *r* in 2015 and  is the growth rate of **p** in year *t*. See Tables S4 and S5 in the Supporting Information (SI) for more details of parameters **p** and **q**_(*t*)_.

In addition, we calculated the capital-embodied CF **g** as **g** = **e**_(*t*)_**L**_(*t*)_**y**_(*t*)_ + **H**_(*t*)_. Here, vector , where *e*_*j*_ is the carbon emission per unit production in sector *j* in year *t*, and vector , where *h*_*j*_ is the direct carbon emission from sector *j* of final demand in year *t*.

The 2015 Japanese input–output table consisting of 390 industrial sectors (*i* = 1···390, *j* = 1···390) defines the initial economic factors (**L**_(*t*)_and **y**_(*t*)_) in eq 1. The data for matrix **R** were obtained from the authors’ previous studies.^27,36^ For vectors **e**_(*t*)_ and **H**_(*t*)_, we used the Embodied Energy Emissions Intensity Data for Japan Using Input–Output Tables (3EID).^37,38^

### 2.3 Model Formulation

In our optimization model, the objective function is aimed at minimizing the impact of material flow constraints on consumption patterns and other economic and social structures. Based on this concept, the constraints are formulated. Initially, we set the objective function to be minimized to the rate of change in per-household consumption from year *t*–1 to year *t* (the first term in eq 3). Then, we set constraints such as material reduction targets, labor availability, export fluctuation limits, food demand security, production capacity, and intergenerational equity (eqs 4–9). However, when these constraints were incorporated, some scenarios resulted in infeasible solutions as the material reduction targets became more stringent toward 2050. To ensure that the 2050 material reduction targets were always met, we introduced a dummy sector to absorb factors that hinder the convergence of the calculations. First, we defined per-household consumption within the dummy sector (the second term in eq 3) to induce production that sustains the workforce for each year. Next, we allowed export fluctuations to exceed the lower bounds that we had set by defining a dummy export fluctuation term (the third term in eq 3). Finally, we defined a dummy output quantity that allowed output to exceed the total value of output in 2015 (the fourth term in eq 3). The newly introduced second through fourth terms were assigned penalty terms relative to the original objective function (the first term). While the first term represents the squared rate of change, the second term uses the squared value of the change, and the third and fourth terms apply a penalty by multiplying positive changes by 1000 times. The sum of all terms (eq 3) was used as the objective function in this model.
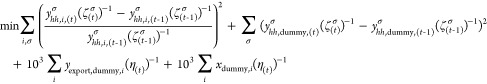
3

The first term in eq 3 indicates that the difference in household commodity consumption (*i* = 1···390) per capita depends on the age of the householder, denoted by σ (σ = 1: under 24, 2:25–34, 3:35–44, 4:45–54, 5:55–64, 6:65–74, 7: over 74). The objective function is set to minimize the rate of consumption change for each product, with the goal of preserving the consumption preferences. Here, *y*_*hh*,*i*,(*t*)_^σ^ represents the consumption per household of sector *i* by attribute σ. (ζ_(*t*)_^σ^)^−1^ denotes the average number of members in a household (family size) by attribute σ. The second term indicates induced production that sustains the workforce each year. *y*_*hh*,dummy,(*t*)_^σ^ represents per household consumption in the dummy sector; in the objective function, it minimizes the changes from year *t*–1 to year *t*. This prioritizes household consumption, which minimizes the rate of change, suppressing the generation of final demand in the dummy sector (in other words, a penalty is imposed on the generation of demand in the dummy sector). The third term indicates the allowance of export fluctuations to exceed the lower bounds we had set. The variables *y*_export,dummy,*i*_ are dummies for the decrease in export of sector *i*. The fourth term indicates a dummy output quantity that allows output to exceed the total value of output in 2015. *x*_dummy,*i*_ represents dummies for the increase in total output capacity of sector *i*. η_(*t*)_ represents the total population in year *t*; a penalty of 1000 times is set for per capita decreases in export and increases in total output. By formulating the optimization model as a penalty problem, we were able to identify optimal solutions that achieve the 2050 material reduction targets in all scenarios considered in this study. A list of the decision variables, parameters, and sets used in the model is given in Table S6 in the SI.

The constraints with respect to *y*_*hh*,*i*,(*t*)_^σ^, *y*_*hh*,dummy,(*t*)_^σ^, *y*_export,dummy,*i*_, and *x*_dummy,*i*_ in eq 3 are subject to the constraints given in eqs 4–9.

Equation 4 shows the constraint based on the material use reduction target. T represents the total material inflow (domestic material inputs + cyclical use) from household consumption and exports in year *t* when the material reduction target is achieved. To calculate *F*_R,(*t*),house,*i*_ and *F*_U,(*t*),house,*i*_, ***y***_house,(*t*)_ is obtained by multiplying *y*_*hh*,*i*,*t*_^σ^ and *y*_*hh*,dummy,(*t*)_^σ^ by the number of households for each generation in year *t*, and then summed.
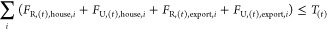
4

Equation 5 is the employment constraint. *W* represents the workforce in year *t*. To calculate W, the proportion of the labor force per working-age population in 2015 is multiplied by the working-age population in year *t*.  represents the employment coefficient per total production for sector *i* and the dummy sector. The employment coefficient for the dummy sector is set as the average value across all sectors. The dummy sector is defined as a sector that does not consume materials or emit GHGs. In year *t*, the dummy sector is subject to penalties in the objective function (eq 3), which suppresses its production. However, when the employment created by the existing sectors falls short of the required workforce, production occurs in the dummy sector.
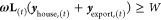
5

Equation 6 is the export constraint. γ_upper,*i*_ and γ_lower,*i*_ represent the maximum rate of increase and decrease rate in exports for sector *i*, respectively. These changes in exports are calculated based on the 5-years export growth rates from 2000 to 2015, and the maximum increase and decrease rates over a 5-years period are set. *y*_export,dummy,*i*_ represents the reduction amount when it is necessary to suppress exports by more than the predefined maximum decrease.
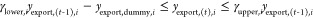
6

Equation 7 is the food demand constraint. *y*_*hh*,food,(*t*),*i*_^σ^ is the per-household consumption expenditure related to demand *i* for food in year *t*. In this model, it is assumed that per-household consumption related to food demand should be maintained at the 2015 level. This ensures that food-related materials are secured as a minimum requirement.
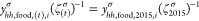
7

Equation 8 represents a constraint on the total output. ***X***_(*t*),*i*_ represents the production amount of sector *i* in year *t* induced by household consumption and exports, and it is set not to exceed 2015 total output *x*_2015,*i*_. In other words, it assumes that the production capacity of production facilities remains constant from 2015. However, if production beyond the production capacity is necessary, it is indicated as *x*_dummy,*i*_.
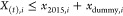
8

Equation 9 promotes equitable reduction in material use across generations. It calculates the per capita MF using the per capita household consumption for each generation based on eqs 1 and 2 and then computes the change rate of per capita MF, *S*_*i*_^σ^, from the base year, *t*–1, for each attribute.
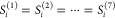
9

### 2.4 Scenario Definition

Four scenarios were defined: business as usual (BaU), demand adjustment (DA), material flow transition (MFT), and innovative technology penetration (ITP). First, the BaU scenario is described below

#### 2.4.1 BaU Scenario

In the BaU scenario, only changes in population are considered while keeping the supply chain structure fixed at the 2015 level with MF and CF calculated. Following the method outlined by Shigetomi et al.,^39^ the National Survey of Family Income and Expenditure,^40^ household consumption data from JIOT for 2015 and the projected population by household and generation in Japan from 2020 to 2040^41^ were used. The consumption per person with attribute σ in 2015 (*y*_*hh*,*i*,(2015)_^σ^(ζ_(2015)_^σ^)^−1^) was calculated and then multiplied by the future household population with attribute σ (θ_(*t*)_^σ^), as shown in eq 10, to estimate the future household consumption in the BaU scenario (*y*_house,*i*,*(t)*,BaU_^σ^).
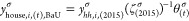
10

Therefore, household consumption, i.e., final demand, changes in response to demographic shifts across generations, and, consequently, GDP also fluctuate. Based on these data, per-household consumption for each age group every 5 years from 2020 to 2040 was estimated. For the years 2045 and 2050, for which household population forecast data were not available, it was assumed that the per-household consumption composition from 2040 would remain the same, and the number of households in each age group was adjusted proportionally to the projected total population for those years. This allowed us to estimate per-household household consumption and age-specific household consumption for 2045 and 2050.

#### 2.4.2 Decarbonization of the Energy Sector and the Material Reduction Target for Carbon Neutrality

For the other three material reduction scenarios, this study envisions two strategies for achieving a carbon-neutral society: the decarbonization of the energy sector (ES) and the establishment of material reduction targets. The ES strategy sets the GHG emission intensity for the electricity sector from Japan’s future energy mix in 2030 and 2050.^42,43^ Linear estimates were applied for other periods. It should be noted that electrification of energy derived from fossil fuels is not considered in this model except for the industrial use of next-generation vehicles assumed in the material reduction scenarios discussed later. For detailed information on the ES strategy, please refer to the SI.

The material reduction target was estimated based on the target of material flow indicators^23^ for the year 2025. Specifically, the reduction rate in total material input required to meet the 2025 targets for resource productivity (GDP/domestic material input (DMI)) and the cyclical use rate (the amount of cyclical use (CU)/total material input (DMI + CU)) is determined. In 2015, resource productivity was 382 thousand yen/t, and the cyclical use rate was 15.9%.^23^ The 2025 targets were set at 490,000 yen/t for resource productivity and 18% for the cyclical use rate. Achieving these targets, assuming a constant GDP, requires an annual decrease of 1.2% in the total material input (DMI + CU). Therefore, we set a standard reduction rate of 2% per year as the target for annual total material input, which achieves the 2025 targets. In addition, ambitious material reduction targets of 3 and 4% per year were set. The rationale for assuming a constant future GDP is that Japan’s real GDP has been nearly flat from 2015 to recent years.

#### 2.4.3 Material Reduction Scenarios for Achieving a Carbon-Neutral Society

The three material reduction scenarios aimed at achieving a carbon-neutral society introduce the aforementioned ES strategy and material reduction targets, as described below

##### 2.4.3.1 DA: Demand Adjustment Scenario

The focus in this scenario is on decarbonizing the energy sector (ES strategy) as a technological option with the aim of achieving material reduction targets solely through demand adjustments.

##### 2.4.3.2 MFT: Material Flows Transition Scenario

In this scenario, based on the material efficiency strategy (MES) highlighted by UNEP-IRP^7^ as key sectors for reducing material-driven GHG emissions in the automotive and housing sectors, the following strategies are assumed

(1) MES for next-generation automobiles.
  This strategy consisted of (1) the transition to next-generation vehicles and lightweight design and (2) the extension of lifetime of next-generation vehicles. For item (1), the share of new vehicle sales for each vehicle type after 2020 and the lifetime of the existing stock are used to estimate the total number of vehicles in circulation in 2050. These estimates are based on the calculations of Kito et al.,^44^ taking into account the expected sales of new cars and the aging of the existing vehicle stock after 2020. Specifically, the stock of next-generation vehicles in 2050 is about 90% (see also Tables S2 and S3 in the SI). In addition, based on the sales shares of different vehicle types in year *t*, the input coefficients for the automotive sector in that year can be estimated. The input coefficients for hybrids and EVs are obtained from the IO table for the analysis of a next-generation energy system^45^ and that for next-generation light vehicles by Pauliuk and Heeren.^46^ Regarding strategy item (2), among the vehicles sold, only the next-generation vehicles have their lifetime extended by 10 years (about 60%). From this extension, it is assumed that the fixed capital consumption of the automotive sector will decrease by 34% in 2050. As the lifetime of vehicles is extended, maintenance and repairs are expected to increase. Therefore, this scenario assumes that the input coefficients for the automobile maintenance sector increase in line with the decrease in fixed capital consumption for automobiles for each year.
(2) Lifetime extension for buildings (residential and nonresidential).
  Referring to the assumptions made by Pauliuk and Heeren,^46^ it is assumed that by 2050, the lifetime of all domestic buildings (residential and nonresidential) will be extended by 90%. As a result, fixed capital consumption for buildings in 2050 is expected to decrease by 47%. From this extension of building lifespans, it is assumed that construction and repairs will increase. For the construction and repair sector, the intermediate input and fixed capital formation are each assumed to increase by 20% in 2050.
(3) Improvement of cyclical use rate. It
  is assumed that strategies to enhance circularity, such as “Enhanced end-of-life recovery and recycling” and “Recovery, remanufacturing, and reuse of components”,^7^ will be comprehensively introduced across industries. The target for the circularity rate in 2025 is set at 18%, while the actual rates were 15.9% in 2015 and 16% in 2020.^23^ Therefore, in this scenario, it is assumed that from 2020 onward, the circularity rate will improve by 2% every 5 years. Additionally, with the implementation of this strategy, it is assumed that the recovery rate of circular resources involving natural resource inputs will also increase. By 2050, the circularity rate is assumed to reach 28%, which means that the recovery rate of circular resources will increase to a sufficient level to achieve this value.

These strategies aim to achieve increases in material efficiency and circularity in the respective sectors. The more detailed data and parameter settings underlying each strategy in the model are provided in the SI. In terms of improving circularity in this model, it is assumed that the amount of cyclical use recovered from waste increases without additional emissions (energy use), meaning that resources can be recovered from waste more efficiently.

##### 2.4.3.3 ITP: Innovative Technology Penetration Scenario

This scenario involves the introduction of innovative technologies in the material production sector, leading to a reduction in the direct GHG emissions from material production. Table S7 lists the material production sectors targeted in this scenario and the assumed innovative technologies.^43^ The GHG emissions per unit production of these sectors in 2050 are defined based on the assumption that the introduction of these technologies results in a 50% reduction in the emission coefficients of each sector compared to 2015.

### 2.5 Limitations

#### 2.5.1 Optimization Model

This model’s optimal solution and pathway toward 2050 represent one of the possibilities for achieving a carbon-neutral society—reducing the material flow while avoiding drastic changes in the objective function, which is household consumption. It is important to note that this is not only the optimal solution but also one among several options for achieving a carbon-neutral society. Furthermore, the optimization model in this study seeks to minimize the changes in household consumption dynamically over time; however, the quantified final demand, material use, and GHG emissions are outcomes representing snapshots for individual years. Therefore, the results obtained through this 5-years interval optimization might differ from those generated with a different time frame (for instance, annual optimization). Moreover, the optimization focuses on minimizing the objective function for each individual period without addressing the minimization of the cumulative change in household consumption throughout the entire period, specifically until 2050. However, considering the objective of this study, to examine how material management targets based on GHG emission reduction contribute to the construction of a carbon-neutral society, we believe this optimization model suffices to achieve this objective.

#### 2.5.2 Stability of Supply Chain Structure in IO Analysis

In this IO model, the technical coefficients **A**_(*t*)_^*d*^ representing the supply chain structure for production and fixed capital formation, excluding sectors considering technology adoption and material efficiency strategies (ES, MFT, and ITP scenarios) defined in Section 2.4, are fixed at their 2015 values. Hence, secondary changes in industrial structures—such as the electrification of heat demand anticipated due to the decarbonization of power in the ES scenario or the material transformation toward low-carbon materials (substituting metal components with low-carbon plastics) due to the decarbonization of material production in the ITP scenario—are not captured. Regarding heat demand, Japan’s carbon-neutral roadmap^43^ suggests using decarbonized electricity for refining decarbonized fuels like hydrogen. Direct electrification of heat demand is limited because of the optimal utilization of existing gas supply facilities. Due to insufficient literature providing supply chain information related to decarbonized fuel production and electrification of heat demand as well as the absence of specific target values, these aspects could not be incorporated into the model. Similarly, information about decarbonized material production is limited. Additionally, in this study’s ITP scenario, it is assumed that “the penetration of innovative technology in material production may not progress at a sufficient pace”. Hence, low-carbon materials are presumed to be solely used in traditional applications, without considering substitution for materials used in other products or capital due to the limitations in progressing innovative technology adoption in material production.

## 3 Results

### 3.1 GHG Emission Reductions through Material Reduction Targets and Carbon-Neutral Transition Scenarios

**Figure 1:**
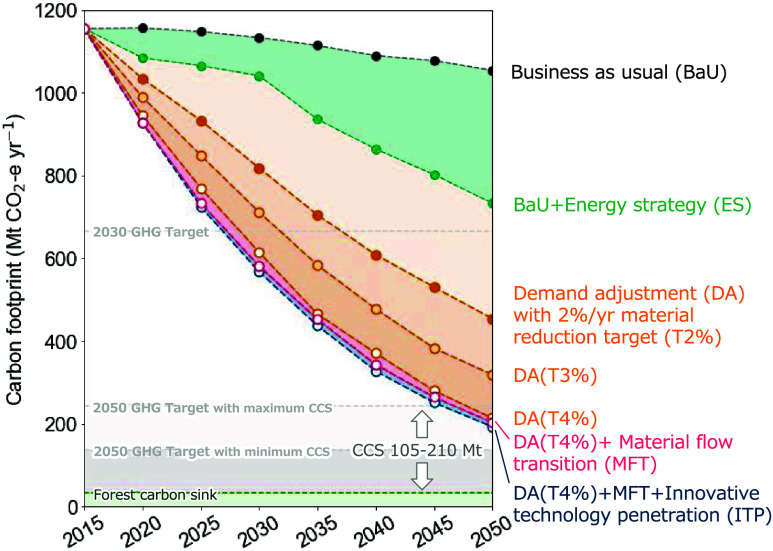
Carbon-neutral pathways through material flows transition in Japan, 2015–2050. GHG emissions induced by domestic household consumption, fixed capital formation, and export are calculated as carbon footprint. The line color represents each carbon-neutral scenario: demand adjustment (DA), material flow transition (MFT), and innovative technology penetration (ITP). All carbon-neutral scenarios involve electricity decarbonization and a material reduction target. DA aims to achieve material reduction targets solely through demand adjustments. MFT aims to increase material efficiency and circularity based on a material efficiency strategy.^7^ ITP involves the introduction of innovative technologies in the material production sector. The marker face color indicates the material reduction targets: T2% (2% annual reduction), T3% (3% annual reduction), and T4% (4% annual reduction). The CCS deployment amount was obtained from the CCS Long-Term Roadmap Review Committee;^47^ the forest carbon sink amount is from the Forestry Agency’s planned values.^48^ The carbon sequestration levels were set based on the proportions of household consumption, fixed capital formation, and exports in total GHG emissions in 2015.

Figure 1 shows the five scenarios used in the study. In these scenarios, a reduction in GHG emissions is achieved through a combination of material reduction targets and transition scenarios for a 2% reduction target (T2%), a 3% reduction target (T3%), a 4% reduction target (T4%) with DA, and a 4% reduction target (T4%) with DA plus MFT or with DA plus MFT and ITP. GHG emissions are calculated as the carbon footprint (CF) induced by final demand, which includes domestic household consumption, fixed capital formation, and exports.

The BaU scenario represents emission reductions due to population changes. Japan’s total population is expected to decrease from 120 million in 2015 to approximately 100 million by 2050. However, the emission reduction resulting from this population decline is less than 10%. Even with an electricity sector that is 100% decarbonized, the emission reduction would be only 300 Mt of CO_2_e per year in 2050, meaning that over 700 Mt of CO_2_e per year of GHGs (approximately 63% of the 2015 level) would continue to be emitted.

To achieve the climate targets, it is necessary to increase the material reduction target to T4%. With T4%, all scenarios (DA, MFT, and ITP) achieve their GHG emission reduction targets by 2050 with a significant amount of CCS. In the DA scenario, achieving a carbon-neutral society requires an 88% maximum planned CCS deployment. By implementation of the strategies of the MFT scenario, the carbon storage requirement of the CCS can be reduced to 83%. Furthermore, with the introduction of innovative technologies assumed in the ITP scenario, the CCS requirement drops to 79%, reducing the dependence. Because the 4% material reduction target already reduces the amount of materials used in society, the impact of material efficiency strategies in the MFT scenario and the introduction of material decarbonization technologies in the ITP scenario is very limited.

### 3.2 Material Flows Structure under the Material Reduction Target

**Figure 2:**
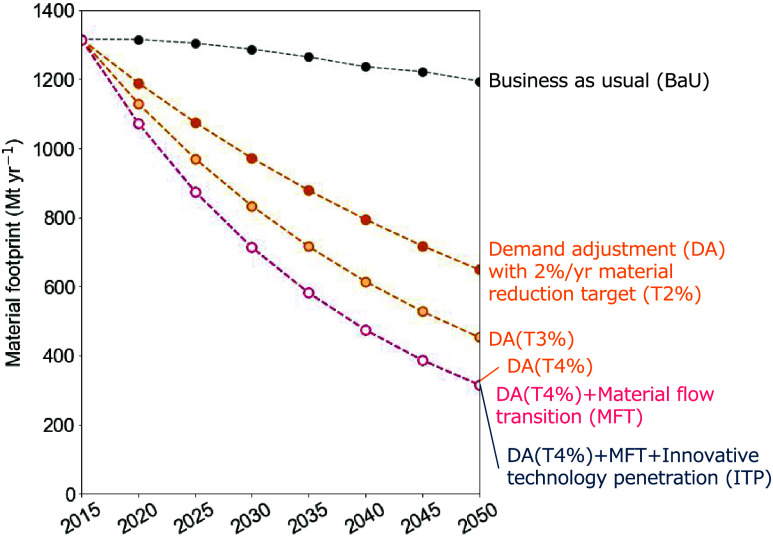
Material footprint in the carbon-neutral scenarios in Japan, 2015–2050. The use of material induced by domestic household consumption, fixed capital formation, and export is calculated as the material footprint. The line color indicates the carbon-neutral scenario: demand adjustment (DA), material flow transition (MFT), and innovative technology penetration (ITP). The marker face color indicates the material reduction targets: T2% (2% annual reduction), T3% (3% annual reduction), and T4% (4% annual reduction).

Setting material reduction targets significantly alters the material flows’ structure. Figure 2 illustrates the trend of material footprints (MFs) for T2%, T3%, and T4% with DA and T4% with MFT. In DA (T2%), the MF in 2050 decreases to approximately half (648 Mt) compared to that in the BaU scenario. In the ambitious DA (T4%), the MF in 2050 is 315 Mt, a quarter of the BaU scenario. As shown in the results of DA(T4%) and DA(T4%) + MFT, because they impose constraints on the total material input, there is no difference in the total amount of MF between DA and MFT when the material reduction target values are the same.

**Figure 3:**
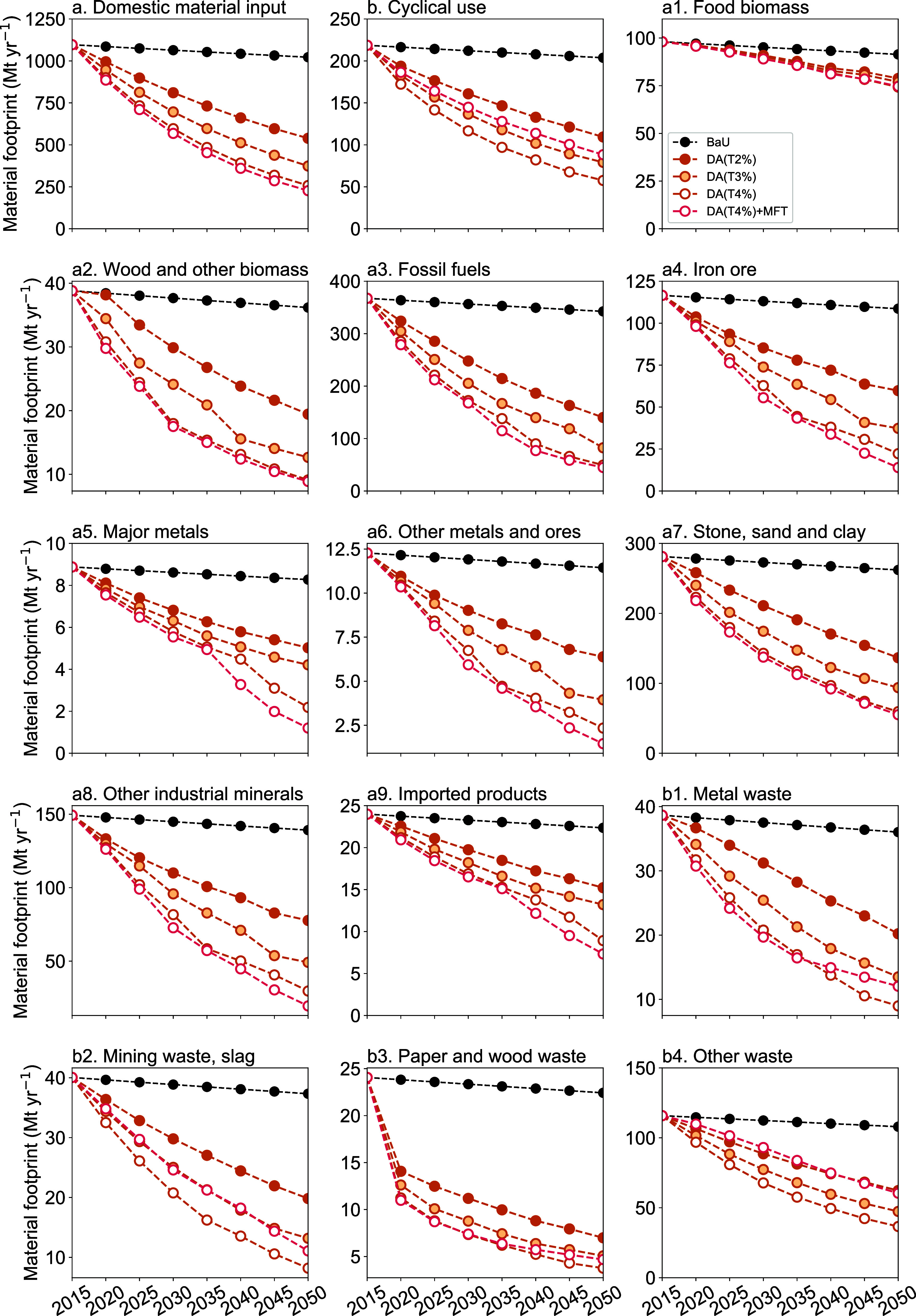
Trends in material footprint in the carbon-neutral scenario by material category in Japan, 2015–2050. (a) Domestic material input shows the total amount of natural resources (a1–a8) and imported products (a9); (b) cyclical use shows the total amount of cyclical use material (b1–b4).

However, the composition of material flows differs significantly between DA and MFT. In the MFT scenario, strategies for improving cyclical use rates are assumed, resulting in a decrease in the domestic material inputs representing inputs such as natural resources (Figure 3a) and an increase in the flow of cyclical use (Figure 3b). In the optimization model used in this study, constraints are set to maintain the final demand for food (Section 4.2), ensuring that food biomass is secured in both the DA and MFT scenarios (Figure 3a1). When broken down by other material categories, inputs of metal resources, such as iron ore and major metal, as well as other mineral resources, decrease significantly (Figure 3a4,a5,a6, and a7). On the other hand, cyclical use such as mining waste and other waste (e.g., rubble) increases (Figure 3b2 and b4). In this way, the MFT scenario maximizes the utilization of cyclical materials by improving the recycling rate, leading to a reduction in the amount of natural resources. However, even with the maximum use of cyclical resources, the constraints on material flow are significant. Compared to the BaU scenario, the MFT scenario requires a decrease of natural resources to about 15–30% and cyclical resources to 20–55%. Of these, fossil fuels, iron ore, other metals and ores, and other industrial minerals are constrained to less than 15%.

**Figure 4:**
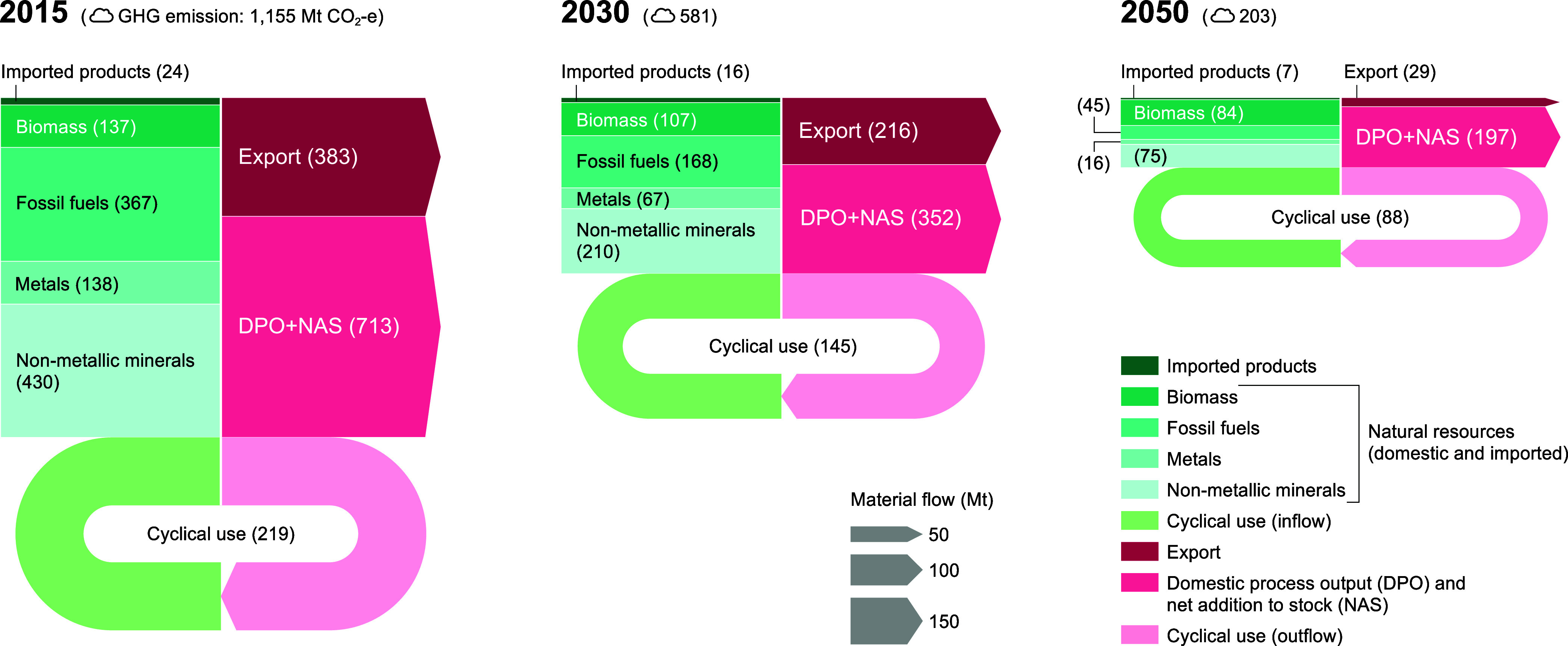
Material flow structure in the carbon-neutral society achieved by the material flows’ transition scenario (DA(T4%) + MFT) in Japan in 2050. All flows are shown to scale in Mt/year; material inflows are shown in green, and material outflows are shown in red.

Figure 4 illustrates the material flows required to achieve carbon neutrality in the DA(T4%) + MFT scenario. While biomass, constrained to meet food demand, maintains the material flow, nonbiomass material flows are decreased by 80–90%. Despite an increase in recycling rates from 16 to 28% due to MFT strategies, domestic process output and net addition to stock (DPO + NAS) decreased by 70%. Since introducing new natural resources is not an option, increasing DPO + NAS requires dismantling and reusing existing stocks. As it becomes more difficult to secure the necessary materials domestically, exports cannot be maintained at more than 90%. Under a material flow consistent with a carbon-neutral society, there may be a trade-off between prioritizing domestic stocks and continuing exports.

### 3.3 Impact of Material Reduction Targets on Economic Structure

**Figure 5:**
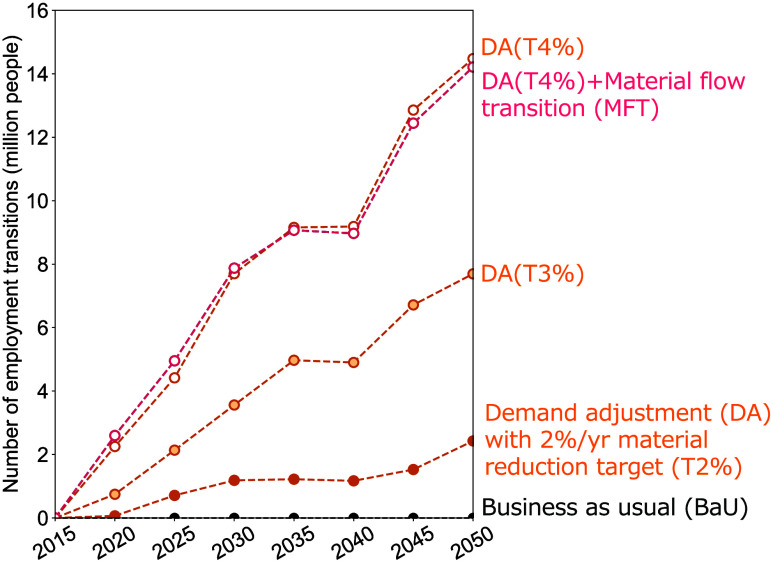
Number of employment transitions required in the carbon-neutral scenarios in Japan, 2015–2050. “Employment transitions” refers to the number of workers who need to transition to new industries because they can no longer maintain employment in their existing industries. The number of transitions is calculated using the final demand in the dummy sector and the ω_dummy_, as indicated in eq 5.

As material flows decline, production activities are expected to stagnate, resulting in an inability to maintain the necessary employment in existing industries. In the scenario where material flows reach 54% of BaU levels in 2050 (DA(T2%) in Figure 2), it is projected that 2 million workers will be unable to find employment in existing industries, necessitating employment in new industries (Figure 5). This represented approximately 3.8% of the workforce at that time. This potential can be seen as the employment transition to new industries required in a carbon-neutral society for the adoption of innovative technologies, CCS, and transformation of material flows.

However, as the material reduction targets become more ambitious, the required number of employment transitions accelerates. In the DA (T3%) scenario, where 38% of BaU material use is allowed, employment transitions of 8 million workers are required (4 times greater than in DA (T2%)). With a 4% reduction, limiting material use to 26% of BaU, carbon-neutral conditions could be achieved, but more than 14 million workers (7 times as many as in DA (T2%)) would be unable to find employment in existing industries (Figure 5). This is close to 30% of the workforce in 2050.

## 4 Discussion

### 4.1 Establishment of Material Flow Targets Consistent with a Carbon-Neutral Society

A mere extension of the past rate of improvement, that is, a material reduction of 2% per year, will not achieve a carbon-neutral society by 2050 even if 100% decarbonized electricity is available. On the other hand, a material reduction of 4% per year will result in the GHG emission reductions necessary for a carbon-neutral society by 2050. This indicates the significance of setting numerical targets that consider the material flow structure in a carbon-neutral society rather than simply maintaining the current rate of improvement. The importance of target-setting also holds true for the circulation rate, which needs to be nearly doubled by 2050 in order to reach the carbon-neutral goal. Achieving a 4% per year material reduction means that the amount of material available for use will be reduced to a quarter of the amount in the BaU scenario; in other words, it would require a 4-fold increase in the material efficiency of consumption. This will require efforts far beyond the MF reduction target discussed in the latest Fundamental Plan for a Sound Material-Cycle Society (a 5% reduction from current levels by 2030).^23^ Simply put, the change to material flow will require a response on the demand side, as well as on the supply side. Thus, attention should be paid to the relationship between target-setting and consumption, and the behavioral changes and specific lifestyle shifts required of consumers to meet the target will need to be identified and promoted.

In shrinking material use, there is a trade-off between domestic stocks and exports. Prioritizing domestic resource use naturally limits the resources available for export. Currently, Japan’s exports are dominated by material-intensive products such as passenger cars, transportation equipment, steel products, petroleum refinery products, and integrated circuits. The results suggest that Japan’s export strategy also needs to be restructured under carbon-neutral conditions.

Looking at the details, the required efficiency levels for natural resource use are even more stringent and vary significantly by resource type. Specifically, wood and stone resources would require a 4-fold and 5-fold increase in efficiency, respectively. However, fossil fuels and ferrous and nonferrous metals would require more than a 7-fold increase in resource efficiency to reach BaU levels. This indicates that the fuel and steel sectors are key not only in terms of reducing GHG emissions^49−54^ (through the adoption of decarbonization technologies) but also in terms of material flow efficiency. These materials provide essential functions for energy, mobility, and infrastructure in modern society.^55,56^ Therefore, there is an urgent and specific need to explore material efficiency strategies, including product and capital concentration, product life extension, material substitution, and resource conservation, to provide these functions with minimal use of natural resources. To sustain society while dramatically reducing the flow of natural resources, the effective use of existing stocks is key.^57^ Developing technologies and business models that allow social functionality to be maintained with minimal material flow for stock maintenance will be essential.

Although innovative, specific technologies for decarbonizing materials’ production, such as hydrogen-reducing steelmaking and carbon-recycling cement and concrete, are being discussed, and plans for their implementation and numerical targets are being established. Many carbon-neutral roadmaps, including Japan’s, focus heavily on the development of innovative decarbonization technologies for materials. However, discussions of material efficiency strategies—which are equally challenging to implement—remain underdeveloped. Material efficiency strategies also require detailed examination of the technologies and policies needed as well as the formulation of roadmaps for their implementation. For example, a 4-fold improvement in material efficiency based solely on the strategies proposed in this study would require the development of technologies that significantly extend the life of buildings and next-generation vehicles many times over, as well as technologies that enable next-generation vehicles to be manufactured using less than half the materials required today. In addition, technologies that enable services that promote the intensive use of materials, such as moving from the current Japanese norm of one car per household to shared services that reduce the average to 0.5 cars per household, are also critical. Furthermore, given the increase in the flow of cyclical use due to improved circularity, it will also be necessary to simultaneously advance energy efficiency improvements in recycling and its decarbonization.

Of course, material efficiency strategies are not limited to those considered in this study. Various combinations of strategies^58^ could achieve material efficiency improvements. However, without support for these efforts with the development of enabling technologies and the design of policies, progress in material efficiency will remain slow. Even Japan, which has legally mandated material flow targets,^23^ has shown that these targets need to be much more ambitious. Such a setting of ambitious material targets can then serve as a driver for achieving carbon neutrality.

In addition, a framework is needed to enable industries and companies to voluntarily contribute to these targets. For carbon emissions, disclosure is increasingly becoming a requirement for corporate listing and financing.^59^ Extending such existing mechanisms and regulations to material flows would create incentives for industries to manage them more effectively. In addition, improving both the efficiency and sufficiency^60^ of material use is critical for consumers. It is also necessary to develop technologies and business models that support this shift. Just as carbon footprints are becoming more visible through apps,^61^ visualizing consumers’ material use can play an important role in driving behavioral change. Efforts to raise awareness and promote changes in consumption habits are key to advancing material efficiency.

### 4.2 Transition in Employment from Material-Intensive Work to Efficient Use and Circulation of Materials

To realize future material flow based on minimized material production and high material efficiency and circulation rates, a significant shift away from employment in material-intensive sectors will be essential. Our results show that shrinking material use in 2050 to between half (for the 2% target) and a quarter (for the 4% target) of BaU levels will require the creation of new jobs for up to 14 million people (30% of the 2050 workforce) to maintain the current Japanese employment rate. Even if the employment transitions were initiated immediately, this would require the transformation of 550,000 jobs per year from 2025 to 2050 (approximately 1% of the total labor force).

The key here is how to create the necessary employment in technologies and services to accelerate the improvement of material efficiency and circulation rate. In concrete terms, current jobs in material-intensive industries will need to transition to industries that either technologically eliminate the need for materials or promote lifestyle changes that require less material use (such as renting, sharing, reusing, and adopting service-based models^62^).

Alternative strategies to secure employment might include simply reducing the workweek from 5 days to 4, which could increase employment by 25%, albeit with a reduction in per capita wage levels. In such a case, creating a scheme to raise the value-added of material circulation, which is at the heart of CE, or strategies for planned economic contraction, such as degrowth,^63−65^ would need to be explored.

In any case, the transitions described above are clearly not on the continuum of the current social development. Policy formulation and implementation will take considerable time. It will be too late to begin the consideration of such policies if we wait until it becomes clear that the introduction of innovative technologies is not progressing as expected.

### 4.3 Decrease in the Impact of Innovative Material Decarbonization Due to Reduced Material Use

The diffusion of innovative material decarbonization technologies will be essential for both the continuation of current material-intensive lifestyles in Japan and the promotion of carbon neutrality. Even if 100% of electricity generation is zero-emissions by 2050, GHG emissions will remain at approximately 63% of their 2015 level. Furthermore, even given a gradual reduction in the GHG emission intensity of material industries (an approximately 1.7% reduction per year from 2020) or a reduction in emission intensity to 50% of the 2015 level, achieving carbon neutrality remains a distant expectation with BaU material use levels (see Supporting Figure 1 in the SI). There is little doubt that the transformation of material flows and the curtailment of material use are imperative. This finding is consistent with decarbonization through reduced material use, as suggested by the literature on low demand and demand-side measures.^13,14^ At the same time, this suggests that the choice of material decarbonization technologies will become more critical.

We also find that the greater the reduction in material use, the smaller the carbon emission reductions resulting from the introduction of material decarbonization technologies. From another perspective, prioritizing the allocation of money, technology, and workforce to material flow transformation with an ambitious reduction target has the potential to further delay and make the diffusion of innovative technologies more uncertain; however, this does not constitute a risk to carbon neutrality. In the remaining years toward a carbon-neutral society, a critical decision must be made regarding whether to bet heavily on innovative decarbonized materials or to prioritize technologies and policies that significantly improve material efficiency in all industries that use materials in the form of products and fixed capital^34,66^ and promote resource-saving, material-efficient lifestyles.^67,68^

## Data Availability

All data and code used in this study have been deposited on GitHub (http://github.com/shohata-data/cn_mft-jp). Permanent references to the data are also accessible through the Zenodo repository.^69^

## Supporting Information Available

Methodological details of scenario analysis; additional results; other data (PDF)

## Notes

Correspondence and requests for materials should be addressed to S.H.
