# Supplementary material for: Material Efficiency and Circularity Goals to Achieve a Carbon-Neutral Society by 2050
Source: Environ Sci Technol. 2025 Mar 20;59(12):6025–36. doi: 10.1021/acs.est.4c08719 (PMC11966779; doi:10.1021/acs.est.4c08719)
Supplement: Supplementary file 1 — es4c08719_si_001.pdf [file es4c08719_si_001.pdf]

Supporting Information

Material efficiency and circularity goals to achieve a carbon-neutral society by 2050

By

Sho Hata*, Keisuke Nansai, Yosuke Shigetomi, Minami Kito, Kenichi Nakajima

DOI: 10.1021/acs.est.4c08719

Contents

Definition of energy strategy for decarbonization

Definition of material flows transition scenario

Definition of cyclical use material

Potential to reduce GHG emissions through innovative technology penetration in the BaU material use

Supplementary Tables S6-S8

(14 pages, 8 tables, 1 figure)

* Corresponding author: Sho Hata

Address: Social Systems Division, National Institute for Environmental Studies, 16-2 Onogawa, Tsukuba 305-8506, Japan

Email: hata.sho@nies.go.jp

Definition of energy strategy for decarbonization

Energy strategy for decarbonization (ES)

As part of the decarbonization strategy for the energy sector, the reduction of emission coefficients in the electricity sector due to the proliferation of renewable energy is incorporated into the model. For the decarbonization of the electricity sector, the Fundamental Energy Plan [1] and the 2030 and 2050 projected energy mixes in Japan [2] served as the primary references. The energy mixes in 2030 and 2050 were set as follows:

Table S1: Energy mix in the electricity sector for the years 2015, 2030, and 2050.

Type of power plants	2015	2030	2050
Fuel power plant	40.6%	1%	0%
Coal power plant	25.7%	19%	0%
Natural gas power plant	23.3%	20%	0%
Renewable power plant	10%	38%	60%
Nuclear power plant	0.4%	22%	40%

For other periods, a linear change in the energy mix between 2015 and 2030 and between 2030 and 2050 is assumed. Based on the assumed energy mix, the input coefficients for the electricity sector, the direct GHG emissions per unit production from the electricity sector, and the direct inputs of fossil fuels for the electricity sector in year t were set. The input coefficient of the electricity sector in 2015 was separated into thermal power generation and other generation based on the 2015 energy mix. The input coefficient for the electricity sector $\mathbf{A}_{electricity,(t)} = (a_{i,electricity,(t)})$ for each year using the energy mix for year t is then estimated according to the following equation:

$$\mathbf{A}_{electricity,(t)} = \nu_{(t)}^{(1)} \mathbf{A}_{non-renewable,(t)} + \nu_{(t)}^{(2)} \mathbf{A}_{renewable,(t)} \qquad (S\text{-}1)$$

Here, $\nu_{(t)}^{(1)}$ and $\nu_{(t)}^{(2)}$ represent the composition of non-renewable energy power plants and renewable energy power plants in year t, respectively.

For direct GHG emissions per unit production, the GHG emissions by fuel type in the electricity sector in 2015 is obtained from 3EID [3,4], setting GHG emissions for coal, fossil fuels, and gas-fired power generation. GHG emissions for each type of thermal power generation are reduced based on the changes in the energy mix since 2015 and the direct GHG emissions for each year in the electricity sector are estimated. Regarding the direct inputs of fossil fuels, the direct inputs for coal, crude oil, refined oil, and natural gas in the electricity sector are decreased based on the 2030 and 2050 energy mixes. Furthermore, for fixed capital consumption, the proportion of renewable energy is increased and that of other power plants is decreased based on the energy mix.

Definition of material flows transition scenario

1) MES for next-generation automobiles

Transition to next-generation vehicles and lightweight design

In this study, the share of new car sales by vehicle type after 2020 and the lifespan of the existing stock are used to estimate the total number of automobiles in circulation up to 2050. These estimates are based on the calculations made by Kito et al. [6], taking into account the expected sales of new cars and the aging of the existing stock of vehicles beyond 2020.

Table S2: Proportion of new car sales by vehicle type.

	GV	HV	EV	Light-HV	Light-EV
2015	100%	0%	0%	0%	0%
2030	33%	58%	9%	0%	0%
2050	0%	11%	14%	33%	42%

Table S3: Stock proportion by vehicle type.

	GV	HV	EV
2015	93.4%	6.0%	0.1%
2020	85.5%	14.3%	0.2%
2025	75.7%	23.7%	0.6%
2030	64.3%	33.5%	2.2%
2035	49.5%	43.5%	7.0%
2040	33.8%	50.0%	16.4%
2045	20.8%	50.9%	28.3%
2050	11.4%	48.8%	39.8%

Based on the sales proportions of different vehicle types in year t, the input coefficients for the automotive sector in that year can be estimated. For the year 2015, it is assumed that the input structure represents gasoline vehicles (GV). The input coefficients for hybrid-vehicle (HV) and EV are obtained from the input-output table to analyze a next-generation energy system, as created by Washizu and Nakano [7], and are adjusted to reflect the sales proportions of each

vehicle type in year t. Additionally, based on the estimations by Pauliuk and Heeren [8], it is assumed that by 2050, Light-HV and Light-EV will account for 75% of vehicle sales and will have a weight that is 80% of their weight in 2015. With these considerations, the input coefficients for the automotive sector in t $\mathbf{A}_{automobile,(t)} = \left(a_{i,automobile,(t)}\right)$ can be shown as follows:

$$\mathbf{A}_{automobile,(t)} = \rho_{(t)}^{(1)}\mathbf{A}_{\mathrm{GV},(t)} + \rho_{(t)}^{(2)}\mathbf{A}_{\mathrm{HV},(t)} + \rho_{(t)}^{(3)}\mathbf{A}_{\mathrm{EV},(t)} + \rho_{(t)}^{(4)}\mathbf{A}_{\mathrm{Light-HV},(t)} + \rho_{(t)}^{(5)}\mathbf{A}_{\mathrm{Light-EV},(t)} \qquad (S\text{-}2)$$

Here, $\rho_{(t)}^{(1)}$ to $\rho_{(t)}^{(5)}$ represent the sales proportions of new cars for each type (GV, HV, EV, Light-HV, Light-EV) in year t.

Lifetime extension of next-generation vehicles

Among the vehicles sold, only next-generation vehicles have their lifetime extended by 10 years (about 60%). The stock of next-generation vehicles in 2050 is approximately 90% (Table S3). By extending their lifetime by 60%, it is assumed that the fixed capital consumption of the automobile sector in 2050 will decrease by 34%. The rate of decrease in fixed capital consumption for year t is set linearly. As the lifetime of automobiles is extended, an increase in maintenance and repairs is expected. Therefore, this scenario assumes that the input coefficients for the automotive maintenance sector increase in line with the fixed capital consumption depletion rate for automobiles in each year, reflecting the increase in automobile repairs.

2) Lifetime extension for buildings (residential and non-residential)

Referring to the assumptions made by Pauliuk and Heeren [8], it is assumed that by 2050, the lifetime of all domestic buildings (residential and non-residential) will be extended by 90%. As a result, fixed capital consumption for buildings in 2050 is expected to decrease by 47%. On the other hand, with the extension of building lifespans, it is assumed that construction and repairs will increase. For the construction and repair sector, the intermediate input and fixed capital formation are each assumed to increase by 20% in 2050. The increase rate for year t is set linearly.

3) Improvement of cyclical use rate

In this scenario, it is assumed that strategies to enhance circularity, such as “Enhanced end-of-life recovery and recycling” and “Recovery, remanufacturing, and reuse of components,” will be comprehensively introduced across industries. The target for the circularity rate in 2025 is set at 18%, while the actual rates were 15.9% in 2015 and 16% in 2020. Therefore, in this scenario, it is assumed that from 2020 onward, the circularity rate will improve by 2% every 5 years.

Additionally, with the implementation of this strategy, it is assumed that the recovery rate of circular resources involving natural resource inputs will also increase. By 2050, the circularity rate is assumed to reach 28%, which means that the recovery rate of circular resources will increase to a level where the circularity rate reaches 28%. In other words, for all circular resources, the recovery rate should be at least 28/72 = 0.39. For circular resources that already exceeded this recovery rate in 2015, their recovery rate is maintained until 2050. For other circular resources, a linear increase in the recovery rate is assumed between 2015 (where the rate is set at 0.39) and 2050.

Note that for paper waste, where the generation rate exceeded 1 for pulp inputs in 2015, the generation rate is set to 1 for all periods. For other circular resources, the rate is set at 0.39 for 2050, and the rate linearly increases for the interim periods.

Definition of the generation rate of cyclical use

In the model, the capital-embodied MF of cyclical use (s) in year t is estimated from the input of natural resources using Equation (4-2). The following lists show the generation rate of cyclical use in year *t* calculated by the generation rate of cyclical use *s* in response to the input of natural resource *r* in 2015, $\mathbf{p}$, and the growth rate of $\mathbf{p}$ in year *t*, $\mathbf{q}_{(t)}$.

Table S4: List of the generation rate of cyclical use in year *t*, $\mathbf{q}_{(t)}\mathbf{p}$, from 2015 to 2050 by material category of cyclical use.

Material category of cyclical use	2015	2020	2025	2030	2035	2040	2045	2050
Cinders/soot	0.057	0.10	0.15	0.20	0.25	0.29	0.34	0.39
Sludge	0.25	0.27	0.29	0.31	0.33	0.35	0.37	0.39
Waste oil	0.23	0.25	0.28	0.30	0.32	0.34	0.36	0.39
Waste acid/alkali	0.19	0.22	0.25	0.27	0.30	0.33	0.36	0.39
Waste plastics	0.31	0.32	0.33	0.35	0.36	0.37	0.38	0.39
Paper waste	1.0	1.0	1.0	1.0	1.0	1.0	1.0	1.0
Wood waste	0.31	0.32	0.33	0.35	0.36	0.37	0.38	0.39
Animal and vegetable residue/food waste/kitchen waste	0.26	0.28	0.30	0.32	0.34	0.35	0.37	0.39
Metals	0.94	0.94	0.94	0.94	0.94	0.94	0.94	0.94
Glass and ceramic waste	0.65	0.65	0.65	0.65	0.65	0.65	0.65	0.65
Mining waste, slag	0.24	0.26	0.28	0.30	0.32	0.35	0.37	0.39
Other	0.24	0.26	0.28	0.30	0.33	0.35	0.37	0.39

Table S5: List of the growth rate of $\mathbf{p}$ in year *t*, $\mathbf{q}_{(t)}$, from 2015 to 2050 by material category of

cyclical use.

Material category of cyclical use	2015	2020	2025	2030	2035	2040	2045	2050
Cinders/soot	1.0	1.8	2.6	3.5	4.4	5.1	6.0	6.8
Sludge	1.0	1.1	1.2	1.2	1.3	1.4	1.5	1.6
Waste oil	1.0	1.1	1.2	1.3	1.4	1.5	1.6	1.7
Waste acid/alkali	1.0	1.2	1.3	1.4	1.6	1.7	1.9	2.1
Waste plastics	1.0	1.0	1.1	1.1	1.2	1.2	1.2	1.3
Paper waste	1.0	1.0	1.0	1.0	1.0	1.0	1.0	1.0
Wood waste	1.0	1.0	1.1	1.1	1.2	1.2	1.2	1.3
Animal and vegetable residue/food waste/kitchen waste	1.0	1.1	1.2	1.2	1.3	1.3	1.4	1.5
Metals	1.0	1.0	1.0	1.0	1.0	1.0	1.0	1.0
Glass and ceramic waste	1.0	1.0	1.0	1.0	1.0	1.0	1.0	1.0
Mining waste, slag	1.0	1.1	1.2	1.3	1.3	1.5	1.5	1.6
Other	1.0	1.8	2.6	3.5	4.4	5.1	6.0	6.8

Potential to reduce GHG emissions through innovative technology penetration in the BaU material use

Supplementary Figure 1, which illustrates the results of introducing innovative technologies to the BaU + ES scenario without setting material reduction targets, demonstrates the maximum potential of emission reduction through the introduction of innovative technologies in the material production sector. This ITP scenario shows that it has the potential to reduce GHG emissions by up to 100 Mt. However, what this result indicates is that even with innovative technologies and energy strategies aimed at maximizing GHG emission reduction, the reduction potential is limited to around 50% of the BaU scenario. To achieve a CN society, controlling the use of the materials themselves is necessary.

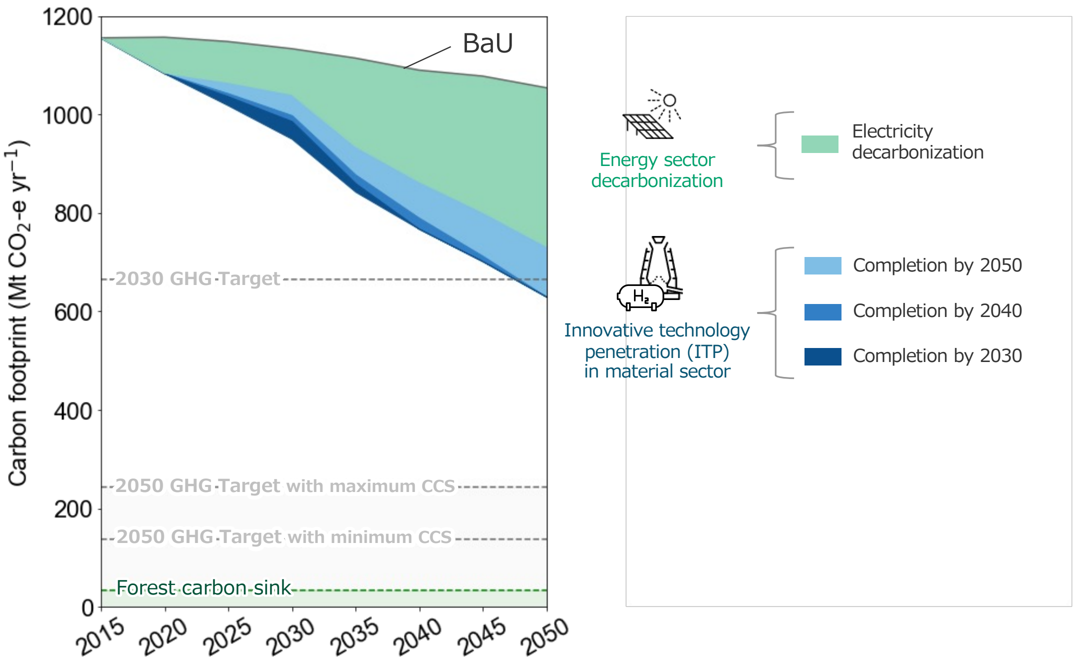

Supplementary Figure 1: Sensitivity analysis showing the impact of different timelines for innovative technology penetration in the BaU scenario

Supplementary tables

Table S6: List of decision variables and parameters and sets used in this model.

Symbol	Unit	Description
$y^{\sigma}_{hh,i,(t)}$	Million yen	consumption per household of sector *i* by attribute σ
$\zeta^{\sigma}_{(t)}$	People	average number of members in household (family size) by attribute σ
$y^{\sigma}_{hh,dummy,(t)}$	Million yen	per household consumption in the dummy sector
$y_{export,dummy,i}$	Million yen	dummy for the decrease in export of sector *i*
$x_{dummy,i}$	Million yen	dummy for the increase in total output capacity of sector *i*
$\eta_{(t)}$	Trillion people	the total population in year *t*
$F_{R,(t),house,i}$	Mt	capital-embodied MF of natural resources in year *t* from household consumption
$F_{U,(t),house,i}$	Mt	capital-embodied MF of cyclical use in year *t* from household consumption
$F_{R,(t),export,i}$	Mt	capital-embodied MF of natural resources in year *t* from export
$F_{U,(t),export,i}$	Mt	capital-embodied MF of cyclical use in year *t* from export
$T_{(t)}$	Mt	material reduction target in year *t*
ω	Million people per million yen	employment per unit production for sector *i* and the dummy sector
$L_{(t)}$	-	capital-endogenized Leontief inverse matrix
$y_{house,(t)}$	Million yen	household consumption in year *t*
$y_{export,(t)}$	Million yen	export in year *t*
W	Million people	workforce in year *t*
$\gamma_{lower,i}$	%	maximum decrease rate in exports for sector *i*
$\gamma_{upper,i}$	%	maximum increase rate in exports for sector *i*
$y^{\sigma}_{hh,food,(t),i}$	Million yen	per-household consumption expenditure related to demand *i* for food in year *t*
$X_{(t),i}$	Million yen	production amount of sector *i* in year *t* induced by household consumption and exports
$x_{2015,i}$	Million yen	total output of sector *i* in 2015
$S^{(\sigma)}_{i}$	%	change rate of per capita MF by attribute σ
$\theta^{\sigma}_{(t)}$	People	Future population by attribute σ

Table S7: List of innovative technologies considered for introduction in the material production sectors and their assumed GHG reduction effect.

Industry	Innovative technologies	GHG reduction effects

Steel	Innovative refining, rolling, and melting methods<br>• Hydrogen reduction ironmaking using carbon-free hydrogen<br>• Expansion of electric furnace processes using carbon-free electricity<br>• Development of innovative heating processes	Reducing emission intensity in the Pig iron sector
Chemical	Carbon-recycled chemicals<br>• Plastic feedstock from artificial photosynthesis<br>• Commercialization of functional chemicals derived from CO_2 as a raw material, as well as chemicals from biomass and waste plastics<br>• Advanced upgrading of naphtha cracking furnaces through carbon-free heat sources and other means	Reducing emission intensity in the Plastic products sector
Ceramics and Soil and Stone	Carbon recycling through concrete and cement<br>• Development and implementation of CO_2-absorbing cement and concrete technology<br>Decarbonization of heat sources (glass, ceramics, etc.)<br>• Transition to heat sources derived from non-fossil fuels such as hydrogen and ammonia	Reducing emission intensity in the Cement sector
Paper	Decarbonization of heat sources<br>• Transition to heat sources derived from non-fossil fuels such as hydrogen and ammonia	Reducing emission intensity in the Pulp, Paperboard, and Paper sectors

Table S8: Category of material use intensity; natural resources and cyclical use.

Major categories	Subcategories
Food biomass	Rice
Food biomass	Wheat
Food biomass	Other cereals

Food biomass	Vegetables, fruit, nuts
Food biomass	Oil seeds
Food biomass	Sugar
Food biomass	Other agricultural products nec
Food biomass	Live animals
Food biomass	Meat
Food biomass	Dairy, eggs, and honey
Food biomass	Fish and aquatic resources
Wood and other biomass	Board and plywood
Wood and other biomass	Fuel wood and charcoal
Wood and other biomass	Lumber and sawn wood
Wood and other biomass	Wood pulp, chips and waste products
Wood and other biomass	Fertilizers
Fossil fuels	Coal
Fossil fuels	Crude oil
Fossil fuels	Refined oil
Fossil fuels	Natural gas and other gaseous hydrocarbons
Fossil fuels	Other fossil fuel products
Iron ore	Iron ores and concentrates
Other metals and ores	Copper ores and concentrates
Other metals and ores	Nickel ores and concentrates
Other metals and ores	Aluminum ores and concentrates
Other metals and ores	Lead ores and concentrates
Other metals and ores	Zinc ores and concentrates
Other metals and ores	Other metal ores and concentrates
Stone, sand and clay	Stone
Stone, sand and clay	Sand and gravel
Stone, sand and clay	Clays
Other imported resource	Cement
Other industrial minerals	Lime

Other industrial minerals	Other minerals
Major metals	Iron and steel
Major metals	Copper
Major metals	Nickel
Major metals	Aluminum
Major metals	Lead
Major metals	Zinc
Other metals and ores	Other non-ferrous metals
Other metals and ores	Gold
Other metals and ores	Other Precious and specialty metals
Other metals and ores	Metals not specified
Imported products	Imported products
Other waste	Cinders and shoot
Other waste	Sludge
Other waste	Waste oil
Other waste	Waste acid/alkali
Other waste	Waste plastics
Paper and wood waste	Paper waste
Paper and wood waste	Wood waste
Other waste	Animal and vegetable residue/ food waste/ kitchen waste
Metal waste	Metals
Other waste	Glass and ceramic waste
Mining waste, slag	Mining waste, slag
Other waste	Other

References

1. Ministry of Economy Trade and Industry. *Fundamental Energy Plan*. (2021).
2. Cabinet Secretariat. *Green Growth Strategy for Achieving Carbon Neutrality by 2050*. (2021).
3. Nansai, K. Embodied energy and emission intensity data for Japan using input–output tables (3EID). *National Institute for Environmental Studies,Japan* http://www.cger.nies.go.jp/publications/report/d031/index.html (2019).
4. Nansai, K., Fry, J., Malik, A., Takayanagi, W. & Kondo, N. Carbon footprint of Japanese health care services from 2011 to 2015. *Resources, Conservation and Recycling* **152**, 104525 (2020).
5. Ministry of the Environment. *Annual Report on the Environment in Japan 2021*. *Government of Japan* (2021).
6. Kito, M., Nakamoto, Y., Kagawa, S., Hienuki, S. & Hubacek, K. Environmental consequences of Japan's ban on sale of new fossil fuel-powered passenger vehicles from 2035. *J Clean Prod* **437**, 140658 (2024).
7. Washizu, A. & Nakano, S. Creation and application of the 2011 input-output table for the next-generation energy system. *Institute for Advanced Social Sciences(IASS) Working Paper Series* **IASS WP 20**, 1–18 (2019).
8. Pauliuk, S. & Heeren, N. Material efficiency and its contribution to climate change mitigation in Germany: A deep decarbonization scenario analysis until 2060. *J Ind Ecol* **25**, 479–493 (2021).
